# Immune-based combination therapy for esophageal cancer

**DOI:** 10.3389/fimmu.2022.1020290

**Published:** 2022-12-15

**Authors:** Huiling Wang, Yufei Xu, Fengli Zuo, Junzhi Liu, Jiqiao Yang

**Affiliations:** ^1^ Laboratory of Integrative Medicine, Clinical Research Center for Breast, State Key Laboratory of Biotherapy, West China Hospital, Sichuan University and Collaborative Innovation Center, Chengdu, China; ^2^ West China School of Medicine, West China Hospital of Sichuan University, Chengdu, China; ^3^ Breast Center, West China Hospital of Sichuan University, Chengdu, China

**Keywords:** esophageal cancer, immunotherapy, chemotherapy, radiotherapy, combination therapy

## Abstract

Esophageal cancer (EC) is an aggressive malignancy raising a healthcare concern worldwide. Standard treatment options include surgical resection, chemotherapy, radiation therapy, and targeted molecular therapy. The five-year survival rate for all stages of EC is approximately 20%, ranging from 5% to 47%, with a high recurrence rate and poor prognosis after treatment. Immunotherapy has shown better efficacy and tolerance than conventional therapies for several malignancies. Immunotherapy of EC, including immune checkpoint inhibitors, cancer vaccines, and adoptive cell therapy, has shown clinical advantages. In particular, monoclonal antibodies against PD-1 have a satisfactory role in combination therapy and are recommended for first- or second-line treatments. Here, we present a systematic summary and analysis of immunotherapy-based combination therapies for EC.

## Introduction

Esophageal cancer (EC) is the seventh most common cancer, with 570,000 cases diagnosed yearly, and the sixth highest cause of cancer-related mortalities, with 509,000 deaths per year worldwide (1). The five-year survival rate for all stages of EC is approximately 20% in China and the USA and only 12% in Europe (2, 3). EC consists of two principal histological subtypes: esophageal squamous cell carcinoma (ESCC) and esophageal adenocarcinoma (EAC). ESCC occurs mainly in the upper and middle parts of the esophagus and is the primary type of EC in Asia and Eastern Europe. EAC occurs mainly in the lower segment of the esophagus, near the stomach or the junction of the gastroesophageal wall, and primarily affects people in Europe and North America (4). Key risk factors of ESCC include smoking, alcohol consumption, hot drinking, and malnutrition. In contrast, risk factors for EAC include smoking, obesity, gastroesophageal reflux disease, and Barrett′s esophagus (5, 6). The incidence of ESCC has been declining while that of EAC has been increasing rapidly, especially in Western men (7).

Traditional therapies for patients with EC include surgery, chemotherapy, chemoradiotherapy, and targeted therapy (8). Traditionally, surgery has been the most common treatment for EC. However, surgery is not suitable for cancer patients diagnosed with distal metastases or advanced stages. American Society of Clinical Oncology (ASCO) recommends chemoradiotherapy or chemotherapy before surgery for most patients with locally advanced EC (9, 10). However, most patients relapse quickly after the initial therapy, with serious adverse events including systemic toxicity and multidrug resistance (8). Thus, novel and effective drugs are needed and expected to improve overall survival (OS).

Immunotherapy is a promising modality for cancer treatment, having anti-tumor effects and increasing the OS of patients with various cancers. However, several clinical trials of immunotherapy for EC indicate that their clinical results remain challenging as a single agent (11). Therefore, a great effort has been focused on developing novel strategies to extend clinical benefits to non-responder populations. One of the strategies is the combination of immune therapy with other systemic therapeutics.

## Immune checkpoint inhibitors therapy in esophageal cancer

The advent of immunotherapy has transformed cancer treatment. As part of its normal function, it can augment or change how the immune system works to curb or slow tumor growth. Several treatment modalities for immunotherapy include immune checkpoint inhibitors (ICIs), cancer vaccines, and adoptive cell therapies (ACTs). However, the major obstacle to immunotherapy is the presence of an immunosuppressive microenvironment, leading to tumor escape from immune surveillance. There are many causes of immunosuppression, including immune checkpoints highly expressed in cancer cells, heterogeneity, and low immunogenicity of tumor antigens (12–15). Immune checkpoint molecules are key co-stimulatory or co-inhibitory signals of the immune response in protecting the host from tissue damage, playing important roles in maintaining self-tolerance and preventing autoimmunity (16–18). However, when tumor cells hyperactivate inhibitory signals, these ligand-receptor pair interactions between tumor cells and T cells negatively regulate T cell activation (19–21). Immune checkpoint molecules include PD1 and CTLA-4 on T-cells and programmed death-ligand 1 (PD-L1) and B7-1/B7-2 on antigen presenting cells (APCs) and tumor cells. Additionally, PD-L1 expression has been studied in several cancers, which can be used to predict the response to ICIs in different cancer types. Immune checkpoint therapy has shown promising clinical responses in several cancers, such as malignant melanoma, head and neck cancer, lung cancer, gastrointestinal adenocarcinoma, and ESCC (22, 23).

**Figure 1:**
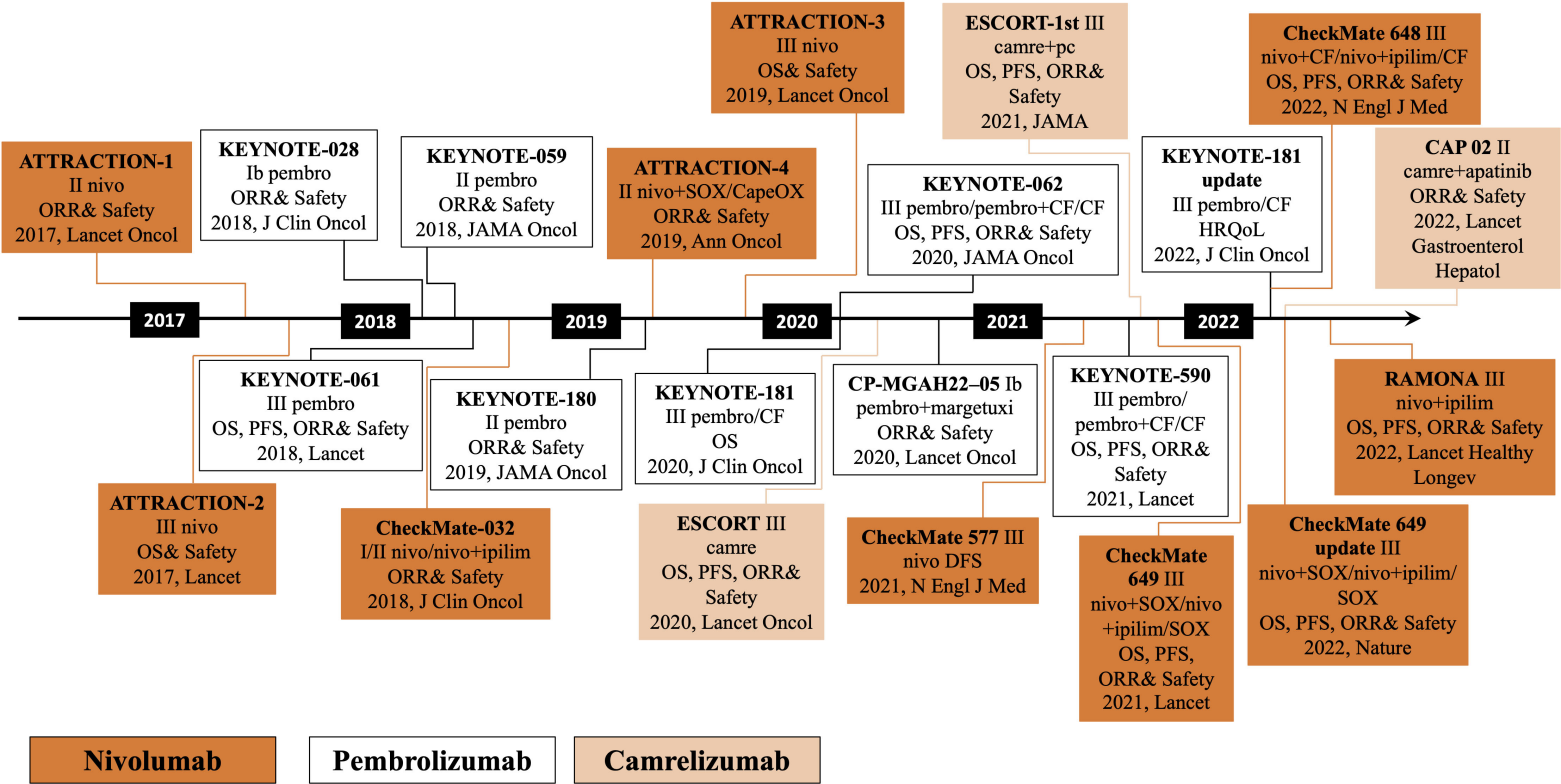
Development of chemoradiotherapy combined with immunotherapy in esophageal cancer. pembro, pembrolizumab; nivo, nivolizumab; camre, camrelizumab; ipilim, ipilimumab; OS, overall survival; PFS, progression-free survival; ORR, objective response rate; DFS, disease-free survival; health-related quality-of-life, HRQoL; S-1 plus oxaliplatin, SOX; capecitabine plus oxaliplatin, CapeOX; paclitaxel plus cisplatin, PC.

The clinical exploration of immunotherapy for patients with advanced EC followed breakthroughs in several areas, including melanoma and lung cancer. The earliest exploration began with the third-line treatment of EC. The ATTRACTION-1 study was the first to explore the efficacy of a PD-1 monoclonal antibody in advanced esophageal squamous cancer (24). In 2018, the Journal of Clinical Oncology published the results of the KEYNOTE-028 study of an EC cohort, followed by the KEYNOTE-180 and KEYNOTE-181 clinical studies of pembrolizumab for third- and second-line treatment of advanced EC, respectively (25–29). Following ATTRACTION-1, the ATTRACTION-3 study of nivolumab versus chemotherapy was conducted as a second-line treatment for advanced ESCC. PD-1 inhibitors, camrelizumab and sintilimab, have advanced the course of domestic PD-1 inhibitors in the second-line treatment of EC. The ESCORT study evaluated the efficacy and safety of camrelizumab compared to the investigator’s choice of chemotherapy in treating patients with advanced or metastatic ESCC who failed first-line chemotherapy. This study is the first randomized, controlled, multicenter phase III clinical study with the largest enrollment of patients with advanced esophageal squamous cancer in China who failed first-line standard chemotherapy. Currently, advanced EC has entered the era of immunotherapy, breaking the treatment bottleneck and providing patients with better options (**Figure 1**).

Pembrolizumab is a humanized IgG4 kappa monoclonal antibody that selectively blocks the interaction of the PD-1 receptor with its receptors PD-L1 or PD-L2. The US Food and Drug Administration (FDA) currently approves this drug for treating breast cancer, hepatocellular carcinoma, melanoma, non-small cell lung cancer, and esophageal or gastroesophageal junction (GEJ) cancer. In KEYNOTE-028, KEYNOTE-180, and KEYNOTE-181 clinical trials, researchers evaluated the safety and efficacy of pembrolizumab monotherapy (27, 30–32). Based on two clinical trials, KEYNOTE-180 and KEYNOTE-181, the FDA approved pembrolizumab as a second-line treatment for advanced or metastatic ESCC. Currently, ASCO recommends pembrolizumab as a first-line drug in combination with chemotherapy for refractory locally advanced or metastatic esophageal and gastroesophageal junction adenocarcinomas and squamous cell carcinomas, regardless of PD-L1 expression.

Nivolumab is a monoclonal anti-PD1 antibody approved by the FDA to treat advanced melanoma, advanced non-small cell lung cancer, advanced renal cell carcinoma, urothelial carcinoma, and squamous cell carcinoma of the head and neck (33–37). The ATTRACTION-1 phase II single-arm trial evaluated the efficacy of nivolumab monotherapy in patients with advanced EC who were refractory to or intolerant of fluoropyrimidine-, platinum-, and taxane-based chemotherapy (24, 38). Based on the ATTRACTION-1 phase II trial, the ATTRACTION-3 phase III trial compared nivolumab monotherapy with taxane monotherapy (paclitaxel or docetaxel) in patients with advanced ESCC after prior fluoropyrimidine and platinum chemotherapy. It concluded that nivolumab monotherapy was one of the promising therapeutic modalities in EC (39, 40). Subsequently, nivolumab was approved by the Pharmaceuticals and Medical Devices Agency and the FDA for advanced EC refractory to fluoropyrimidine- and platinum-based drugs.

Camrelizumab is a humanized anti-PD-1 monoclonal antibody independently developed by China. Phase I clinical trials showed that camrelizumab was well tolerated by patients with advanced solid tumors and showed anti-tumor activity (41–43). In China, camrelizumab has been approved for the treatment of several malignancies, such as Hodgkin’s lymphoma, advanced hepatocellular carcinoma, non-small cell lung cancer (NSCLC), and EC. In addition, camrelizumab exhibited encouraging efficacy in some patients with advanced ESCC in a wider phase I dose-escalation and expansion study (NCT02742935) (44). Currently, camrelizumab is approved as second-line therapy for ESCC in China.

## Cancer vaccine in esophageal cancer

Traditional prophylactic vaccines protect humans from diseases caused by viruses or bacteria by exposing people to weakened or killed germs with preserved immunogenicity but lost antigenicity. Prophylactic vaccines, such as the chickenpox vaccine, are administered to healthy people to avoid disease in the future (45). Therapeutic vaccines are administered to treat existing malignancies. Cancer cells originate from the healthy cells of the host. The process of carcinogenesis is believed to involve the accumulation of somatic mutations by stepwise progression, resulting in cancer cells closely resembling normal cells to a certain extent (46). Besides immune cell exhaustion, the side effects of cancer therapies or aging contribute to the severe debilitation of the immune response (47–49). Therefore, treatment vaccines for cancer face severe challenges because of tumor-induced immunosuppression, immune evasion, and the aging immune system (50, 51). Over the past 40 years, only two therapeutic cancer vaccines have been approved in the United States and the European Union: sipuleucel-T and talimogene laherparepvec. Currently, to improve the effectiveness of cancer vaccines, choosing optimal antigens and highly potent vaccine vectors and quelling tumor-mediated immunosuppression have been described as the most important considerations in the design of therapeutic vaccines.

The application of a therapeutic vaccine for EC focuses on New York esophageal squamous cell carcinoma 1(NY-ESO-1) and melanoma-associated antigen (MAGE-A), both well-known cancer-testis antigens (CTAs) with re-expression in numerous cancer types. Owing to their restricted expression patterns and ability to elicit immune responses, CTAs are promising candidates for cancer vaccines (52, 53). Bujas et al. and Forghanifard et al. analyzed the expression profiles of MAGE-A4 and NY-ESO-1 using immunohistochemistry and relative mRNA expression, respectively. Both showed overexpression of MAGE-A4 and NY-ESO-1 in patients with EC (54–56). Kageyama et al. confirmed the safety and immunogenicity of the CHP-NY-ESO-1 vaccine by comparing the effectiveness of repeated inoculation with 100 µg or 200 µg CHP-NY-ESO-1 (57). Several studies have explored the combined application of cancer vaccines and immune adjuvants. Ishikawa conducted a clinical trial on a CHP-NY-ESO-1 vaccine combined with poly-ICLC and observed that the combination treatment group exhibited better antibody responses than cancer vaccine alone (58). A phase I study of vaccination with NY-ESO-1f peptide combined with Picibanil OK-432 and Montanide ISA-51 in patients with cancers expressing the NY-ESO-1 antigen enrolled six patients with EC and observed an increase in NY-ESO-1 antibody response and CD4 and CD8 T cell response in nine of ten patients (59), indicating the importance of dendritic cell-based cancer vaccines. Since dendritic cells (DCs) are the dominant antigen-presenting cells and strong activators of T cells, numerous studies have investigated the use of peptide-pulsed DCs as cellular vaccines (53, 60, 61). Narita conducted a phase I/II clinical trial in ESCC, demonstrating that anti-tumor immunotherapy with a SART1 peptide-pulsed DC vaccine may not bring clinical and survival benefits (62). However, the vaccine was well tolerated, with acceptable side effects. Several clinical trials have indicated the safety and feasibility of WT1 peptide-pulsed DC vaccinations, with WT1-specific immunity augmented (63, 64). Some trials have also investigated the combination of multiple highly immunogenic human leukocyte Antigen HLA-restricted epitopes of overexpressed CTAs in patients with ESCC, showing promising anti-tumor activity (65–67).

## Adoptive cell therapy in esophageal cancer

ACT, including chimeric antigen receptor (CAR)-and T cell receptor (TCR)-engineered T cell therapies and tumor-infiltrating lymphocytes (TILs), exhibits effective and rapid therapeutic effects on tumors. Typically, modification of T cells is a stepwise process *in vitro*, involving extracting the patient’s T cells; isolating, modifying, and expanding them ex vivo; and then returning them to the patient. Standard gene transfer methods include transient mRNA transfection, lentiviral transduction, and retroviral vector transduction.

The CAR molecule is major histocompatibility complex (MHC)-independent, and recognition and binding of specific antigens primarily depend on extracellular domains, namely single chain variable fragments (68). Therefore, the tumor immune escape elicited by low human leukocyte antigen (HLA) expression can be avoided. Owing to its potent and long-lasting anti-tumor functions, the clinical response of hematological tumors has shown great success. Genetically engineered autologous CD19 targeted CAR-T cells were the first therapeutic modality approved by the FDA for treating relapsed or refractory hematological malignancies such as lymphocytic leukemia and B-cell lymphoma. In addition, Autologous B-cell maturation antigen-targeted CAR-T cell therapy products for multiple myeloma have shown outstanding anti-tumor activity (69). Nevertheless, despite the remarkable achievements of CAR-T cell therapy in treating liquid tumors, the widespread use of CAR-T cell therapies to treat solid tumors is comparatively modest because of the various roadblocks (69).

Currently, preclinical trials with EC-associated antigens for CAR-T cell therapy targeting HER2, EphA2, MUC1, B7, and H3 are ongoing. Although clinical responses and results are less satisfactory in some solid cancers, Several clinical trials of CAR-T cell therapy against EC are investigating and evaluating (NCT03706326, NCT03740256, NCT03013712, NCT04581473) (70).

An alternative genetically modified T-cell immunotherapy is TCR-T therapy, exhibiting a broader treatment effect. In contrast to CAR-T cells, the TCR-T cell construct is a heterodimer consisting of α and β chains. Antigen recognition by the αβTCR is core to the function of the adaptive immune system (71). Recognition and binding of T cells to antigens depend on the specific matching of TCRs with HLA, resulting in T cells distinguishing rare foreign pMHCs from abundant self pMHC molecules (72–74). Compared with CAR-T cell therapy, TCR-T cell therapy could act on more targeted antigens. In addition to antigens expressed on the surface of cells, intracellular antigens can also be recognized once processed and presented by MHC molecules (75).

Much research on TCR-T cell therapy mainly concentrates on solid tumors (76) rather than liquid tumors, targeting CTAs, including NY-ESO-1, MAGE-A3, and MAGE-A4. Moreover, preclinical and clinical trials of TCR-T-cell therapy for EC are ongoing.

## Immunotherapy in combination with chemoradiotherapy

### PD1/PD-L1 inhibitors

**Figure 2:**
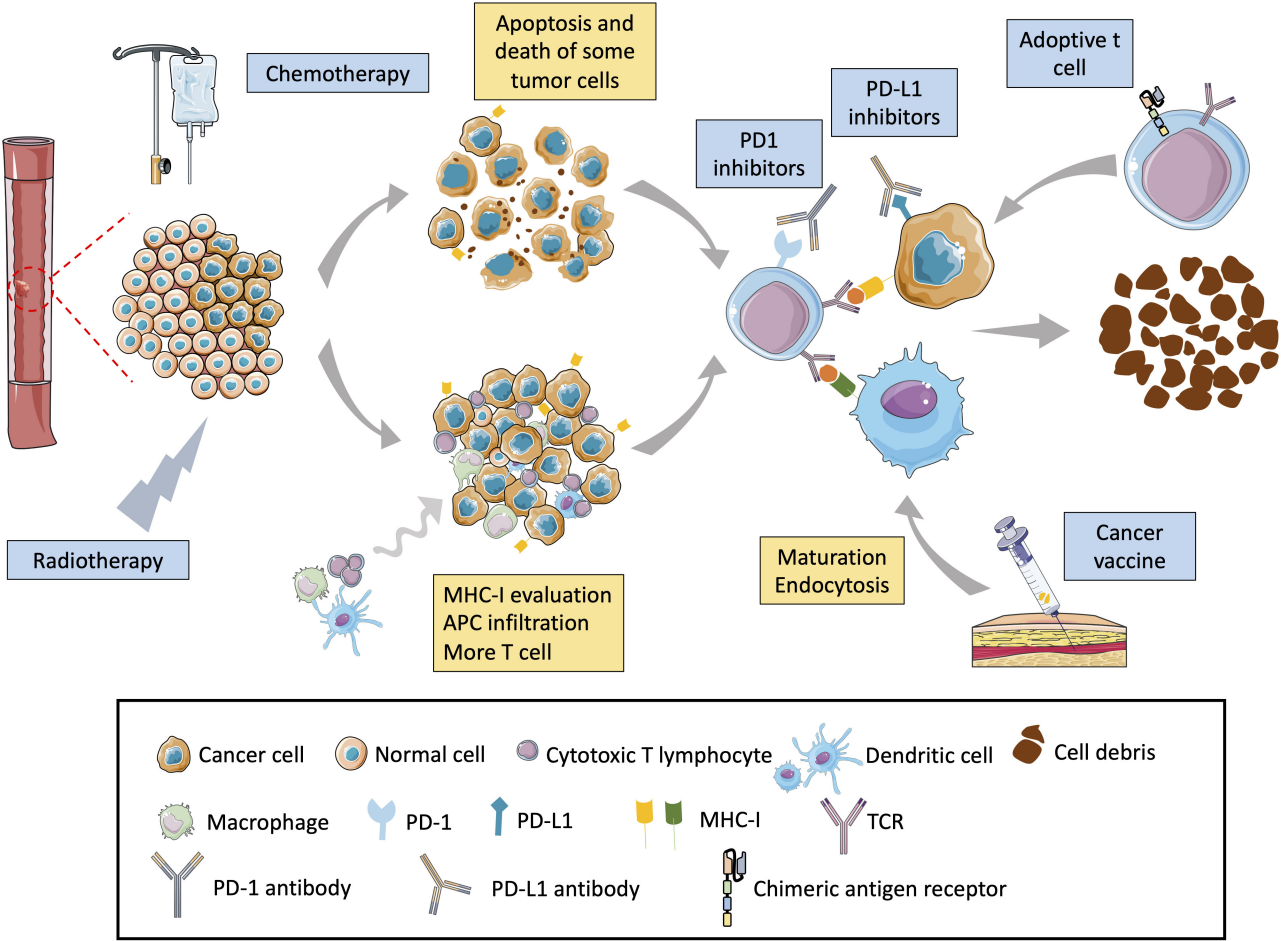
Mechanism of chemoradiation–immunotherapy combination in esophageal cancer. Chemoradiation can directly lead to the death of cancer cells by apoptosis, necrosis, and autophagy, promoting the release of tumor-specific antigens by tumor cells and increasing the chances of immune cells finding cancer cells. Chemoradiation can also directly destroy the DNA of cells, causing cancer cells to produce neoantigens and triggering an immune response. In addition, chemoradiation can upregulate the expression of tumor MHC-I that can better present tumor-specific antigens and enhance tumor visibility by cytotoxic T cells. Radiotherapy can modulate the tumor microenvironment, increase the tumor microenvironment, and promote the migration of cytotoxic T cells to the tumor. Radiotherapy also upregulates the PD-L1 expression level on the cancer-cell surface, enhancing the therapeutic effect of PD-L1 antibodies.

Previous studies showed chemoradiation combined with immunotherapy to have good anti-tumor effects. This combination treatment includes three action mechanisms: (1) Chemoradiation can kill tumor cells, release tumor antigens, and increase the recognition of T cells (77). (2) Chemoradiation can increase the expression of antigens on the surface of tumor cells (78). In addition, studies have shown that chemotherapy increases the molecular expression of MHC-I, thereby strengthening immune system recognition of tumor cells (79). (3) Radiotherapy and chemotherapy destroy the tumor microenvironment and increase T-cell infiltration (80–82). In addition, some studies have shown that radiotherapy has a range of effects, such as activating dendritic cells, reducing the level of regulatory T cells in tumors, expanding the lineage of T cells, and enhancing T cell metastasis (**Figure 2**). The combination of immune checkpoint therapy and chemoradiotherapy has shown promising clinical responses in several cancers, such as malignant melanoma, head and neck, lung, gastrointestinal adenocarcinoma, and ESCC.

Pembrolizumab-based monotherapy can prolong the OS and progression-free survival (PFS) of patients with ESCC, with acceptable treatment-related adverse effects. The FDA has approved pembrolizumab to treat ESCC (PD-L1 CPS ≥ 10). Clinical trials of pembrolizumab and chemotherapy combination for the treatment of EC have achieved significant results, and more clinical trials are underway.

The KEYNOTE-059 study, a multi-cohort, phase II, non-randomized clinical trial, compared the effects of pembrolizumab in combination with chemotherapy to those of chemotherapy alone as first-line treatment in patients with advanced gastric or gastroesophageal junction adenocarcinoma (**Table 1**). The study enrolled 25 and 31 patients, respectively. This trial demonstrated encouraging anti-tumor activity as a first-line treatment and acceptable safety. However, because the sample size was small, it needs to be validated in a larger population. The phase III randomized clinical trial KEYNOTE-062 evaluated the efficacy and safety of first-line treatment in patients with untreated, advanced gastric/GEJ (G/GEJ) cancer with a PD-L1 CPS of ≥ 1 (**Table 1**). The study enrolled 763 patients randomly divided into pembrolizumab (n = 256), pembrolizumab plus chemotherapy (n = 257), or chemotherapy (n = 250) treatment groups. This clinical trial demonstrated clinically meaningful OS of pembrolizumab in patients with untreated, advanced G/GEJ cancer with PD-L1 CPS of ≥ 1, especially in those with PD-L1 CPS of ≥ 10 and high microsatellite instability (MSI) tumors. However, the benefits of pembrolizumab and chemotherapy combination were not superior to those of chemotherapy alone, regardless of PD-L1 CPS of more than 1 or 10 (83–85). These data are consistent with those of the KEYNOTE-061 study, confirming the utility of PD-L1 high expression and suggesting pembrolizumab for frontline therapy (86). The results provide a reliable basis for further research on pembrolizumab in combination with chemotherapy.

**Table 1:** Clinical Trial of Immunotherapy in Combination with Chemoradiotherapy.

NCT Number	Phase	Role	Conditions	regime	mOS(m)	mPFS(m)	ORR (%)	mDFS(m)
KEYNOTE-059 (NCT02335411) cohort 2	II	first-line	advanced G/GEJ adenocarcinoma	PENBRO+CHEM	13.8	6.6	60	NA
KEYNOTE-062 (NCT02494583)	III	first-line	untreated, locally advanced/unresectable, or metastatic G/GEJ cancer (PD-L1 CPS≥1)	PENBRO+CHEMO (CF/CAP) vs PLACEBO+CHEMO	12.5 vs 11.5	6.9 vs 6.4	48.6vs 37.2	NA
KEYNOTE-590 (NCT03189719)	III	first-line	advanced EC and Siewert type 1 GEJ cancer	PENBRO+CHEMO (CF)vs PLACEBO+CHEMO	12.4 vs 9.8	6.3 vs 5.8	45vs 29.3	NA
ATTRACTON-4 (NCT02746796)	III	first-line	HER2-negative, unresectable advanced or recurrent G/GEJ cancer in Asian	NIVO + CHEMO (SOX/CAPOX) vs PLACEBO+CHEMO	17.45 vs 17.15	10.45 vs 8.34	57 vs 48	NA
CheckMate649 (NCT02872116)	III	first-line	advanced or metastatic G/GEJ, and EAC	NIVO + CHEMO vs CHEMO	14.4 vs 11.1(PD-L1 CPS≥ 5)	7.7vs 6.0(PD-L1 CPS≥ 5)	60 vs 45(PD-L1 CPS≥ 5)	NA
CheckMate 648 (NCT03143153)	III	first-line	advanced ESCC	NIVO + CHEMO(CF) vs CHEMO	15.4 vs. 9.1(PD-L1 CPS≥ 1) 13.2 vs. 10.7	6.9 vs 4.4(PD-L1 CPS≥ 1) 5.8 vs 5.6	53 vs 20(PD-L1 CPS≥ 1) 47 vs 27	NA
NCT03222440	Ib	first-line	locally advanced ESCC	CAMRE+RADIO	16.7	NA	73	11.7
ESCORT-1st (NCT03691090)	III	first-line	advanced or metastatic ESCC	CAMRE+CHEMO (CP) vs PLACEBO+CHEMO	15.3 vs 12	6.9 vs 5.6	72.1 vs 62.1	NA
NIC-ESCC2019 (NCT04225364)	II	neoadjuvant treatment	resectable ESCC	CAMRE+CHEMO (CP)	NA	NA	66.7	NA
ORIENT-15 (NCT03748134)	III	first-line	unresectable, locally advanced recurrent or metastatic ESCC	SINTILI+CHEMO (CP/CF) + PLACEBO+CHEMO	17.2 vs 13.6(PD-L1 CPS≥ 10) 16.7 vs 12.5	8.3 vs 6.4(PD-L1 CPS ≥10) 7.2 vs 5.7	78.8 vs 57.5(PD-L1 CPS ≥10) 75.5 vs 56.9	NA

mOS, median overall survival; mPFS, median progression-free survival; ORR, median objective response rate; mDFS, median disease-free survival; EC, esophageal cancer; GEJ, gastro-esophageal junction; PEMBRO, pembrolizumab; CHEMO, chemotherapy; NIVO, nivolumab; CF, 5-fluorouracil and cisplatin; CAP, capecitabine; SOX, S-1 plus oxaliplatin; CAPOX, capecitabine plus capecitabine; CP, cisplatin plus paclitaxel; CAMRE, camrelizumab; RADIO, radiotherapy; SINTILI, sintilimab; NA, no assessment.

KEYNOTE-590 (NCT03189719) was a randomized, placebo-controlled, double-blind, phase 3 study to evaluate the anti-tumor activity of pembrolizumab plus chemotherapy in comparison with that of chemotherapy alone as the first-line treatment in patients with unresectable, locally advanced, or metastatic EC or Siewert type 1 GEJ cancer (**Table 1**). A total of 749 patients were enrolled in a randomized 1:1 trial of pembrolizumab plus chemotherapy and chemotherapy alone. The KEYNOTE-590 study combined immunotherapy and chemotherapy to derive the advantages of immunotherapy enhanced by synergistic effects. The study showed a significant improvement in median PFS (mPFS), documenting the short-term efficacy of mPFS, objective response rate (ORR), and the long-term effect of median OS (mOS). Thus, the combination of pembrolizumab and chemotherapy can provide a comprehensive survival benefit to patients. Compared with the KEYNOTE-062 and ATTRACTION-4 trials that did not indicate a distinct difference in OS between combination therapy and mono-chemotherapy, the OS in KEYNOTE-590 was significantly improved. This may be due to the sample size differences and the use of post-study anti-tumor agents. By comparing ICIs in combination with chemotherapy as first-line treatment and ICI monotherapy as second- or third-line treatment, more significant survival benefits and higher levels of PD-L1 expression in tumors were observed in the former. This phenomenon could be because previous therapy generated tolerance, immunosuppressive tumor microenvironments, and the synergistic effect of ICIs plus chemotherapy. The limitations of this trial include no separation of adenocarcinoma and squamous cell carcinoma in the study, no stratifying analysis based on PD-L1 status, and unknown HER-2 status (87). Since combining pembrolizumab with chemotherapy in KEYNOTE-590 trials as first-line treatment provided meaningful improvement, FDA approved pembrolizumab plus chemotherapy as first-line therapy for advanced or metastatic esophageal or GEJ cancer.

Definitive chemoradiotherapy is the standard treatment modality for unresectable EC or distant metastasis. Based on previous study, A double-blind, phase III randomized placebo-controlled study KEYNOTE-975 (NCT04210115) currently in progress is evaluating the efficacy and safety of pembrolizumab plus definitive chemoradiotherapy compared to placebo plus definitive chemoradiotherapy as first-line treatment for patients with esophageal carcinoma. The data from this trial have not been reported hitherto.

The FDA approved nivolumab in 2021 for patients receiving neoadjuvant radiotherapy for completely resected esophageal or GEJ cancer with the residual pathological disease (88). In addition, clinical trials of combination chemotherapy for EC have also made significant progress, and the exploration of treatment options for combination chemotherapy is ongoing.

ATTRACTION-4 (NCT02746796) is a randomized, multicenter, double-blind, placebo-controlled, phase II/III clinical trial in patients with previously untreated, unresectable, advanced, or recurrent G/GEJ cancers (**Table 1**). Based on the safety and efficacy of nivolumab plus chemotherapy observed in the phase II trial of ATTRACTION-4 (89), the latest phase III clinical research results were published in The Lancet ontology. A total of 724 patients were randomly assigned to receive nivolumab plus chemotherapy and chemotherapy in a 1:1 ratio. Compared to CheckMate649, this trial was conducted in Asia, including Japan, South Korea, and Taiwan. It demonstrated that the PFS (hazard ratio (HR): 0.68; 98.51% confidence interval (CI): 0.51–0.90; p = 0.0007), ORR, and more durable responses favored nivolumab plus chemotherapy over chemotherapy alone. However, the OS difference was insignificant (HR: 0.90, 95% CI: 0.75–1.08; p = 0.26) in this trial, showing superior OS with the combination therapy in CheckMate649. The disparity between the ATTRACTION-4 trial and CheckMate-649 study could be attributed to differences in subsequent anti-tumor treatment modalities. *Post-hoc* interaction analyses suggested that most of the baseline characteristics were not determinants of treatment outcomes in either ATTRACTION-4 trial or CheckMate-649 study. Furthermore, no new safety issues were observed. Nivolumab combined with chemotherapy showed a manageable safety profile. A limitation of ATTRACTION-4 was the absence of an assessment between the PD-L1 CPS and each endpoint. In the CheckMate-649 and KEYNOTE-062 trials, the survival advantage of nivolumab plus chemotherapy and pembrolizumab plus chemotherapy was more significant in patients with a higher PD-L1 CPS than in those with a lower PD-L1 CPS. In conclusion, nivolumab in combination with chemotherapy has the potential to become a new first-line treatment for Asian patients with HER2-negative, unresectable advanced or recurrent G/GEJ cancers (90, 91).

Compared with the ATTRACTION-4 clinical trial conducted primarily in Asian populations, the CheckMate 649 trial was conducted in different countries with a broader population.

CheckMate649 (NCT02872116) is a randomized, multicenter, open-label, phase III study that was conducted in patients with untreated, unresectable, non-HER2-positive gastric, GEJ, or EAC to evaluate the efficacy and safety of nivolumab-plus-chemotherapy (**Table 1**). Among the 1,581 advanced patients enrolled regardless of PD-L1 CPS expression, 789 patients were treated with nivolumab-plus chemotherapy, and 792 were treated with chemotherapy alone. In all randomly assigned patients or patients with a PD-L1 CPS of ≥ 5, the combination therapy group displayed better OS and PFS than the group subjected to chemotherapy alone. Statistical hierarchical testing suggested that OS and PFS benefit magnitudes were relative to the PD-L1 CPS cut-offs. The higher the PD-L1 CPS cut-off, the greater the OS and PFS benefits. For patients with PD-L1 CPS ≥ 5, evidence for using chemo-immune combination therapy regimens is relatively strong. However, the suitability of patients with a PD-L1 CPS of < 5 for chemotherapy in combination with immunotherapy requires further active exploration and search for relevant biomarkers. Examples include tumor mutational burden (TMB), copy number variant load, and neutrophil-to-lymphocyte ratio. The safety profile of nivolumab plus chemotherapy was acceptable and consistent with known individual treatments. Based on this trial, the FDA approved nivolumab plus chemotherapy as a new standard first-line treatment for previously untreated patients with advanced G/GEJ cancer and EAC (89, 92).

Doki et al. compared the first-line treatment with nivolumab-based therapy in patients with previously untreated, unresectable advanced, recurrent, or metastatic ESCC. The experimental design of CheckMate 648 relied heavily on the success of CheckMate 649. CheckMate 648 (NCT03143153) clinical trial is a randomized phase 3 study that enrolled 970 patients. Patients were randomly divided into three groups in a 1:1:1 ratio to receive nivolumab plus chemotherapy, nivolumab plus the monoclonal antibody ipilimumab, or chemotherapy alone (**Table 1**). This trial demonstrated significant OS and PFS clinical benefits associated with nivolumab plus chemotherapy in patients with the proportion of PD-L1-positive tumor cells to total tumor cells is greater than one percentage. Furthermore, the proportion of patients with numerically higher objective response rates and longer durations of response in the nivolumab plus chemotherapy cohort was larger than those in the chemotherapy alone cohort, among patients with the proportion of PD-L1-positive tumor cells to total tumor cells is greater than one percentage. Additionally, a pre-planned exploratory subgroup analysis showed that tumor cell PD-L1 expression and PD-L1 combined positive score had clinical utility (93).

Camrelizumab has been approved as second-line therapy for ESCC, and its combination with chemotherapy has been approved as first-line therapy for patients with advanced or metastatic ESCC in China. The following is a summary of the main clinical trials of camrelizumab combined with chemotherapy.

A phase Ib study (NCT03222440) investigated the safety and feasibility of definitive radiotherapy plus camrelizumab as a first-line treatment for locally advanced ESCC (**Table 1**). Twenty patients were recruited for the study. The trial demonstrated promising clinical results and an acceptable safety profile. Additionally, predictive biomarkers and specific status and function of T-cell subsets were assessed by analyzing the tumor microenvironment and systemic immune status (94). Based on these preliminary results, a phase III, randomized, double-blind, placebo-controlled study of camrelizumab versus placebo in combination with concurrent chemoradiationin these patients (NCT04426955) was conducted this year.

The ESCORT-1st (NCT03691090) study was a randomized, double-blind, placebo-controlled, phase 3 trial conducted between December 3, 2018, and May 12, 2020 (**Table 1**). This trial enrolled 596 eligible patients from 60 hospitals in China. All patients were randomly assigned (1:1) to the camrelizumab-chemotherapy and placebo-chemotherapy groups. The ESCORT-1st study has three highlights: First, the enrollment criteria are tailored for the Chinese population. As 90% of patients with EC in China are diagnosed with squamous carcinoma, the ESCORT-1st study was designed to include a population of Chinese patients with squamous EC, in line with the actual pathology in China. Second, the paclitaxel plus cisplatin regimen is China’s most widely used chemotherapy for advanced EC. Therefore, the ESCORT-1st study is more suitable for the Chinese scenario than the fluorouracil+cisplatin regimen used in overseas clinical studies for advanced EC. Third, camrelizumab plus chemotherapy showed superior efficacy and safety results than placebo plus chemotherapy. The analysis of this clinical trial data suggested that camrelizumab plus chemotherapy showed better OS and PFS than placebo plus chemotherapy, with an acceptable adverse event profile similar to monotherapy. No new adverse events were identified. Furthermore, the results also showed statistically significant improvements in health-related quality-of-life metrics with camrelizumab plus chemotherapy compared to that with placebo plus chemotherapy. There are also several limitations to the trial, including the absence of correlation between PD-L1 expression status and efficacy of camrelizumab plus chemotherapy and the discovery of predictive biomarkers (95). Overall, there are still many directions for this study worth exploring.

Camrelizumab showed promising anti-tumor activity in ESCC as a first-line treatment, and researchers have explored the effect of camrelizumab as neoadjuvant therapy. The phase II trial NIC-ESCC2019 (NCT04225364) assessed camrelizumab plus chemotherapy in resectable ESCC as a neoadjuvant option in China (**Table 1**). The trial showed the combination therapy’s feasibility, safety, and efficacy and indicated that neoadjuvant chemoimmunotherapy might have a better response in lymphatic metastases than in primary lesions (96). However, further research is required due to the small sample size.

Currently, the NICE-2 (NCT05043688) study is designed as a three-arm, multicenter, prospective, randomized, phase II clinical trial to evaluate the efficacy and safety of camrelizumab plus chemotherapy (IO-CT) and camrelizumab plus chemoradiation therapy (IO-CRT) versus CRT as preoperative treatments for locally advanced ESCC. The primary endpoint is the complete pathological response rate, and secondary endpoints include event-free survival, R0 resection rate, and adverse events. Patient enrollment in this trial started in September 2021. It is still in the recruitment stage (97).

Camrelizumab is a PD-L1 monoclonal antibody independently developed by China, and the current clinical trials related to camrelizumab are mainly conducted in China. Although it is approved for second-line treatment of esophageal cancer in China, its application is still very limited worldwide. In the future, it is worth looking forward to carrying out Camrelizumab-related clinical trials for EC in more regions outside China.

Additionally, sintilimab is a domestic anti-PD-1 monoclonal antibody used in China. It was first approved by the National Medical Products Administration (NMPA) for patients with relapsed or refractory classical Hodgkin’s lymphoma after two or more lines of systemic chemotherapy. Subsequently, the NMPA approved sintilimab in combination with chemotherapy as first-line treatment for NSCLC and in combination with IBI305 as first-line treatment for hepatocellular carcinoma. Therefore, investigators have evaluated sintilimab in combination with chemotherapy for the treatment of EC. ORIENT-15 (NCT03748134) is a global, randomized, double-blind phase III study that evaluated the efficacy and safety of sintilimab combined with chemotherapy versus chemotherapy alone as the first-line treatment in patients with unresectable locally advanced, recurrent, or metastatic ESCC (**Table 1**). At the data cut-off, all 659 patients were enrolled and randomized into sintilimab/placebo plus chemotherapy in a 1:1 ratio. Based on the OS, PFS, and ORR analysis, investigators have suggested that the combination of sintilimab and chemotherapy can be considered a new first-line treatment in patients with advanced or metastatic ESCC (98). However, because this study is ongoing, more evidence of its efficacy and safety profile may be forthcoming, along with a longer follow-up.

### Cancer vaccine

CRT has been widely used to treat patients with irresectable ESCC. Nonetheless, not all patients are resistant to chemoradiotherapy, and many relapse. To date, cancer vaccines have shown promising results in therapy and a manageable safety profile for EC (**Figure 2**). Therefore, combining chemoradiotherapy with cancer vaccines may be an effective way to treat EC.

The purpose of phase I clinical study (NCT00632333) of multiple-epitope peptide vaccines combined with CRT was to evaluate its safety and efficacy. As a result, all 11 patients with unresectable chemo-naïve ESCC showed peptide-specific cytotoxic T lymphocyte responses to at least one of the five peptide antigens during vaccination. After the 8th peptide vaccination in combination with CRT, 54.5% (6 of 11) achieved CR, along with 45.5% (5 of 11) showing PD. All patients tolerated the combination therapy well and did not experience serious adverse effects (99). However, the number of patients in this study was small, and there was no control group. Therefore, it is difficult to explain the practical effects of a cancer vaccine in combination with CRT.

Fujiwara et al. conducted a phase I/II open, non-randomized, single-arm clinical trial (UMIN 000000669) between July 1, 2007, and July 1, 2011, to investigate the safety and efficacy of labeled DC combined with systemic chemotherapy for EC. Five patients were enrolled in this study. This study demonstrated that the accumulation of DC in primary tumors injected with labeled DC did not migrate to the lymph nodes from primary tumors. No DC accumulation was observed elsewhere. Additionally, there were no changes in the antibody titers of the 28 tumor antigens analyzed by the enzyme-linked immunosorbent assay. However, the clinical responses of the five enrolled patients were absent (100).

Wang et al. conducted a clinical trial to observe the efficacy of a combination of radiotherapy and dendritic cells loaded with apoptotic heat-shock EC cell antigens. There was a remarkable increase in the expression of serum IL-2, IL-12, and IFN-γ and the proportion of IFN-γ^+^CD8^+^T cells in the treatment group, compared to that in the baseline and control groups (all P < 0.05). The 1- and 2-year survival rates improved with vaccination. Only two patients had a mild fever. This clinical trial recruited 40 patients randomly divided into experimental and control groups in a 2:1 ratio. However, because of the study’s small sample size, patients being in an early-stage EC, and follow-up time being relatively short, more multicenter trials are necessary for combining radiotherapy and DC-based cancer vaccines (101).

To date, most vaccines in combination with chemoradiotherapy against EC are in the developmental stage—the clinical application of combining immunotherapy with tumor vaccines for EC warrants further exploration.

### Adoptive cell therapy

Adoptive cell therapy is also synergistic with chemoradiotherapy (**Figure 2**). Sato et al. conducted two phase-I trials of adoptive γδT cell therapy combined with chemotherapy. One was for treatment-refractory recurrent or metastatic EC (r/mEC) (γδT-monotherapy-P1, UMIN000001419) and the other for r/mEC with no prior systemic therapy (DCF-γδT-P1, UMIN000008097). The results of the 26 γδT-monotherapy patients enrolled suggest no survival benefits and no severe adverse events. Eight patients received docetaxel, cisplatin, and 5-fluorouracil (DCF) chemotherapy plus adoptive γδT cell therapy, and a better clinical response was obtained, similar to that in the DCF mono-chemotherapy previously reported. All treatment-related adverse events were associated with DCF chemotherapy but not with γδT injection. However, this was a phase I study with a small sample size designed to evaluate the safety. Therefore, large, randomized phase 2 controlled studies are warranted (102).

## Combination between immunotherapies

Currently, several clinical trials evaluate the function of ICI monotherapy, such as anti-PD-1/PD-L1 and anti-CTLA-4, in patients with EC. These trials showed favorable results for ICIs as second-line or higher-line therapies, with relatively few side effects. In addition, combination treatment with ICIs has shown promising clinical benefits in some malignant tumors, such as advanced renal cell carcinoma, advanced melanoma, and advanced NSCLC (103–106). However, the role and efficacy of combination therapies with anti-PD-1 and anti-CTLA-4 antibodies in EC is ongoing.

The CheckMate-032 (NCT01928394) study aimed to assess the efficacy and safety of nivolumab and nivolumab plus ipilimumab in patients with metastatic esophagogastric cancer in western countries (**Table 2**). Patients (n = 160) were randomly divided into three groups: nivolumab 3 mg/kg, nivolumab 1 mg/kg plus ipilimumab 3 mg/kg, and nivolumab 3 mg/kg plus ipilimumab 1 mg/kg. The CheckMate-032 study was the first to demonstrate the potential clinical benefits and manageable safety profile of nivolumab and nivolumab plus ipilimumab in patients with chemotherapy-refractory esophagogastric cancer. Based on the efficacy and safety of different doses, the phase III CheckMate-649 study selected NIVO1 + IPI3 for further evaluation. Additionally, investigators explored potential predictive biomarkers, including tumor PD-L1 and MSI status. However, further studies are warranted because of the small sample size (107, 108).

**Table 2:** Clinical Trial of Combination between Immunotherapies.

NCT Number	Phase	Role	Conditions	regime	mOS(m)	mPFS(m)	ORR (%)	mDFS(m)
CheckMate-032 (NCT01928394) cohort2	I/II	adjuvant treatment	chemotherapy-refractory EC in Western	NIVO1+IPI3 vs NIVO3	6.9 vs 6.2	1.4 vs 1.4	24 vs 12	NA
CheckMate-032 (NCT01928394) cohort3	I/II	adjuvant treatment	chemotherapy-refractory EC in Western	NIVO3 + IPI1	4.8	1.6	8	NA
CheckMate648 (NCT03143153)	III	first-line	advanced ESCC	NIVO+IPI vs CHEMO	13.7 vs. 9.1(PD-L1 CPS≥1) 12.7 vs 10.7	4 vs 4.4(PD-L1 CPS≥1)	35 vs 20(PD-L1 CPS≥1) 28 vs 27	NA
RAMONA (NCT03416244)	II	second- line	advanced ESCC (age≥65)	NIVO+IPI	2.7	6.9	NA	NA

mOS, median overall survival; mPFS, median progression-free survival. ORR, median objective response rate. mDFS, median disease-free survival; NIVO, nivolumab; IPI, ipilimumab; IPI1, ipilimumab 1 mg/kg; IPI3, ipilimumab 3 mg/kg; NIVO1, nivolumab 1 mg/kg; NIVO3, nivolumab 3 mg/kg; NA, no assessment.

CheckMate-648 is the largest randomized, global phase III study to date in which ICIs are based on the first-line treatment of advanced ESCC, with a total of 970 patients from multiple countries and regions around the world, including China. In addition, it is the only phase III clinical study evaluating the first-line treatment of advanced ESCC in the first-line dual immune (PD-1 inhibitor + CTLA-4 inhibitor) “de-chemotherapy” regimen; it received widespread attention. The CheckMate648 (NCT03143153) study recruited patients with previously untreated, unresectable advanced, recurrent, or metastatic ESCC who received treatment with nivolumab plus chemotherapy, nivolumab plus the monoclonal antibody ipilimumab, or chemotherapy monotherapy in a 1:1:1 ratio (**Table 2**). The results of nivolumab plus chemotherapy compared with those of chemotherapy alone are presented above. Of note, OS and objective response were significantly better with nivolumab plus ipilimumab than with chemotherapy among patients with tumor cell PD-L1 expression of ≥ 1% and in the overall population. However, the PFS differences between the two groups did not meet the criteria for statistical significance. The incidence of grade 3 or 4 treatment-related adverse events among those who received nivolumab plus ipilimumab was 32% and that for chemotherapy alone was 36%, lower than that of nivolumab plus chemotherapy (47%). The incidence of treatment-related deaths was similar across groups (93).

RAMONA (NCT03416244) is a multicenter, open-label, phase II trial conducted in 34 centers in Germany to investigate the safety of nivolumab and ipilimumab as second-line therapy in elderly patients with advanced ESCC and additional comorbidities (**Table 2**). It enrolled 66 eligible patients between May 2018 and August 2020. The patients received combined nivolumab and ipilimumab therapy and nivolumab alone in a 2:1 ratio. With the median follow-up of 6.8 months (3.4–15.4), the mOS of 7.2 months was significantly improved compared to that in the previous control cohort receiving standard chemotherapy (p = 0.0063). Treatment-related adverse events were observed in 42 patients. Adverse events of grade 3 or worse occurred in 54 (82%) of the 66 patients, and serious adverse events occurred in 45 (68%) patients. Overall, grade 3–5 treatment-related adverse events occurred in 13 (20%) patients, with no difference between the patients who received nivolumab monotherapy (five [23%] of 22) or combination therapy (eight [18%] of 44) (109).

Many experts and researchers believe that double immunotherapy’s adverse reaction rate is lower than that of chemotherapy. Patient tolerance is good, but the serious adverse reactions caused by immunotherapy, such as bone marrow suppression and gastrointestinal reactions, are more difficult to deal with than adverse reactions caused by chemotherapy. For example, some experts believe that the 32% incidence of adverse events of CheckMate 648 grades 3 or 4 is too high. Although the efficacy of double immunotherapy in advanced EC is remarkable, its side effects cannot be denied.

## Immunotherapy in combination with targeted drugs

Targeted drugs have significant efficacy in the treatment of tumors due to their small toxicity and high specific advantages (110–112). Apatinib is a small-molecule anti-angiogenic agent that targets the VEGFR-2 tyrosine kinase. Anti-angiogenic agents can increase the infiltration of immune effector cells into tumors and reprogram the tumor microenvironment through the normalization of the tumor vasculature. The addition of anti-angiogenic agents can enhance cancer immunotherapy. Apatinib is the second agent approved by the China Food and Drug Administration for treating advanced metastatic gastric cancer. Several preclinical and clinical trials have demonstrated its vigorous anti-tumor activity and acceptable safety in advanced gastric cancers and ECs.

A single-arm, open-label, investigator‐initiated phase II study of apatinib monotherapy evaluated its effectiveness and safety profile in patients with unresectable, metastatic EC (113). The phase II clinical trial ESO‐Shanghai 11 evaluated the efficacy and adverse effects of oral apatinib in patients with chemotherapy-refractory ESCC (114). The results of the two clinical trials showed that apatinib monotherapy has the potential to be an efficient and secure second-line or higher treatment for patients with ESCC. Zhao et al. conducted a trial to evaluate the safety and efficacy of apatinib combined with neoadjuvant chemotherapy in patients with locally advanced ESCC compared with chemotherapy alone. They concluded that combination therapy has promising outcomes in the treatment of ESCC (115).

A single-center phase II clinical trial (NCT03603756) investigated the efficacy and safety of camrelizumab plus apatinib in combination with chemotherapy as the first-line treatment (**Table 3**). This trial enrolled 30 patients between August 7, 2018, and February 23, 2019. The primary endpoint ORR was 80% (19/26), achieving the prespecified primary endpoint. Compared to chemotherapy in a previous clinical trial, combination therapy showed encouraging clinical outcomes. Because the mOS between the PD-L1 CPS ≥ 10 and < 10 subgroups was similar, the results of subgroup analysis showed that the clinical effect was not directly associated with PD-L1 CPS. However, since this was a small sample size, single-arm experiment, a larger clinical trial is needed to assess the relationship between PD-L1 expression and clinical response (114, 116).

**Table 3:** Clinical Trial of Immunotherapy in Combination with Targeted Drugs.

NCT Number	Phase	Role	Conditions	regime	mOS(m)	mPFS(m)	ORR (%)	mDFS(m)
NCT03603756	II	first-line	unresectable, locally advanced recurrent or metastatic ESCC	CAMRE+apatinib+ CHEMO(NP)	19.43	6.85	80	NA
CAP 02 (NCT03736863)	II	second-line	advanced ESCC	CAMRE+apatinib	15.8	6.8	34.6	NA
CP-MGAH22–05 (NCT02689284)	Ib-2	first-line	previously treated, HER2-positive GEJ adenocarcinoma	PEMBRO+margetuximab	12.48	2.73	18.48	NA

mOS, median overall survival; mPFS, median progression-free survival. ORR, median objective response rate. mDFS, median disease-free survival; CAMRE, camrelizumab; CHEM, chemotherapy; NP, liposomal paclitaxel plus nedaplatin; PEMBRO, pembrolizumab; NA, no assessment.

The CAP 02 (NCT03736863**)** trial explored the efficacy of camrelizumab in combination with apatinib as second-line therapy for advanced ESCC (**Table 3**). This single-arm, open-label, phase II study was conducted at eight centers in China and enrolled 52 patients. The objective response rate in this study was close to that observed in CheckMate 648 (47%) and KEYNOTE-590 (45%), indicating anti-tumor activity of camrelizumab plus apatinib in ESCC. However, considering the inconsistencies in the conditions of the three clinical trials, this result should be interpreted with caution. *Post-hoc* analyses of the correlation between PD-L1 expression and clinical responses indicated promising clinical responses, regardless of the amount of PD-L1 expressed. However, the mechanism of action of camrelizumab in combination with anti-angiogenic drugs remains unclear. Larger-scale clinical validation is needed (116). Subsequent camrelizumab plus apatinib protocol was designed as a cohort study of follow-up treatment options for patients who failed first-line immunotherapy. It is believed that this study can bring more therapeutic hope to immune-resistant populations and will further confirm the advantages of anti-angiogenesis combined with immune regimens.

Margetuximab, a novel, investigational, Fc-engineered, anti-HER2 monoclonal antibody, offers more effective antibody-dependent cellular cytotoxicity than trastuzumab-mediated innate immune cells. In a phase 1 study of patients with refractory HER2-positive gastroesophageal adenocarcinoma, margetuximab monotherapy resulted in an objective response rate of 10% (two of 20 patients) and enhanced adaptive immunity (117). Anti-HER2 agents have also been reported to increase PD-L1 expression in tumor cells. In preclinical models, synergistic anti-tumor activity has been observed when anti-HER2 therapeutic approaches are combined with anti-PD-1 antibodies.

Therefore, Catenacci et al. conducted a clinical trial to evaluate the safety, tolerability, and anti-tumor activity of margetuximab plus pembrolizumab in previously treated patients with HER2-positive gastroesophageal adenocarcinomas. CP-MGAH22–05 (NCT02689284), a single-arm, open-label, phase Ib–2 dose-escalation, cohort-expansion study, enrolled 95 patients between February 11, 2016, and October 2 (**Table 3**). The median follow-up was 19.9 months. Margetuximab plus pembrolizumab cohort showed manageable safety and tolerability. In addition, no dose-limiting toxicities were observed during the dose-escalation phase (118).

Immunotherapy has become the standard for the first- and second-line treatment of advanced EC. However, the issue of no standard treatment for immune-resistant populations has begun to receive clinical attention. The mechanism of immune resistance is very complex. More studies, such as double immune combination, immune combination anti-EGFR monoclonal antibody, immune combined cyclin inhibitors, and immune combined epigenetic drugs, need to be carried out in the future to increase the effect of combination therapy and bring more clinical benefits to patients.

## Future direction and conclusion

In recent years, clinical trials have emphasized that ICIs combined with chemotherapy or radiotherapy may achieve greater therapeutic effects on various cancers than ICIs alone. For example, immunotherapy has shown encouraging clinical results in the treatment of EC. Immune checkpoint therapy has been rapidly developed for the treatment of EC. For example, based on Keynote-590, the FDA approved pembrolizumab in combination with platinum and fluoropyrimidine-based chemotherapy for patients with metastatic or locally advanced esophageal or GEJ carcinoma who are not candidates for surgical resection or definitive chemoradiation. Based on CheckMate-648, FDA approved nivolumab in combination with fluoropyrimidine- and platinum-based chemotherapy and nivolumab in combination with ipilimumab as the first-line treatment for patients with advanced or metastatic ESCC.

Gastric cancer and EC have high heterogeneity, and there are great differences in the biological characteristics and clinical characteristics of Chinese and Western patients, bringing difficulties to clinical research. For example, in the CheckMate-649 study, the degree of benefit for patients in the Chinese subgroup was significantly higher, showing that patients in different countries and regions can receive different levels of benefits. Therefore, more influencing factors should be considered in the future when conducting a hierarchical analysis. In addition, China has a strong and unmet clinical demand for EC drug treatment, therefore, more research on Chinese patients with EC is needed.

While immune checkpoint-based therapy is promising, only a small proportion of patients benefit from immunotherapy. Therefore, accurate screening of target populations and combination therapy will become the main direction of future research on advanced EC. Further, immune-related side effects cannot be ignored. Another area of research is finding strong predictive and prognostic biomarkers or comprehensive biomarkers to optimize treatment strategies. Many clinical trials have explored PD-L1 positivity, TMB, MSI, and T cell inflammatory gene expression profiles as biomarkers. However, because these trials differ in the chemotherapy backbone, anatomical and histological differences, PD-L1 diagnostic antibodies, and positive definitions of PD-L1, any crossover trial comparison should be made with caution.

Tumor vaccines offer a therapeutic approach that helps to direct the immune system to recognize cancer-associated antigens and achieve anti-tumor effects. Tumor vaccines mainly include whole-cell, molecular, and DC vaccines. Although preclinical and clinical trials on cancer vaccine monotherapy have yielded preliminary results, combining tumor vaccines with other regimens requires further exploration. In addition, the discovery and utilization of new antigens have contributed to the development of cancer vaccines.

Several ACTs have shown promising clinical utility in treating EC with acceptable toxicity. Further research is needed to reduce toxicity and improve the efficiency of this strategy. TILs are important candidates for the ACT and have demonstrated anti-tumor activity in preclinical and clinical studies to treat several solid tumors, including melanoma and ovarian cancer. Researchers have also found that TIL is significantly associated with survival in patients with EC, but current TIL-based studies for treating EC have not been reported.

Immunotherapy-based combination therapies have shown positive effects in EC, and more clinical trials are still underway. Nevertheless, more research is needed to identify new targets and expand immunotherapy to the first line. In addition, identifying better biomarkers to provide prognostic information and guide therapy is critical to the breadth of precision oncology. Further studies on the tumor microenvironment, the molecular mechanisms of response, and resistance to checkpoint inhibitors will also be instructive for immunotherapy.

With the increasing application of combination treatment regimens in the first-line treatment of patients with advanced gastric cancer and EC, further developing a second-line treatment plan after a patient’s treatment fails is still controversial. Clinicians may choose chemoradiotherapy alone or chemoradiotherapy plus anti-angiogenic drugs or continue to use combination regimens. In the future, the provision of more standardized clinical treatments for such patients needs to be studied further. In addition, whether the combination therapy plan of reduction and exemption can move the frontline forward and apply it to the perioperative treatment of patients with early EC also requires corresponding thinking and exploration by oncology clinicians.

## Author contributions

HW contributed to investigation, illustration, writing, and editing. YX, FZ contributed to investigation and writing-review. JL, JY contributed to writing-review and editing. All authors contributed to the article and approved the submitted version.
